# Mortality Statistics, Chicago

**Published:** 1875-10

**Authors:** Ben. C. Miller

**Affiliations:** Sanitary Superintendent


***Chicago Mortality Report for August, 1875.*** Reported by Dr. BEN. C. MILLER, Sanitary Superintendent.

| | |
|---|---|
| Accident, crushed | 3 |
| " drowning | 12 |
| " exhaustion | 1 |
| " fall | 4 |
| " in elevator | 1 |
| " by poison | 2 |
| " railroad | 10 |
| " shooting | 1 |
| " suffocation | 1 |
| Abscesses | 4 |
| " lumbar | 1 |
| " psoas | 1 |
| Apoplexy | 4 |
| Bowels, intussusception of | 1 |
| Brain, anæmia of | 1 |
| " compression of | 1 |
| " congestion of | 7 |
| " hæmorrhage of | 1 |
| " inflammation of | 4 |
| " softening of | 3 |
| Bronchitis | 8 |
| " capillary | 3 |
| Caries of vertebræ | 1 |
| Cancer of breast | 2 |
| " of peritoneum | 1 |
| " of stomach | 6 |
| " of uterus | 5 |
| Child birth | 3 |
| Cholera infantum | 278 |
| " morbus | 3 |
| Colitis | 2 |
| Consumption | 44 |
| Convulsions | 100 |
| " puerperal | 2 |
| Croup | 4 |
| Cyanosis | 2 |
| Debility, general | 12 |
| Deficient development | 2 |
| Diarrhœa | 66 |
| " chronic | 8 |
| Diphtheria | 10 |
| Dropsy, general | 3 |
| " ovarian | 1 |
| Dysentery | 30 |
| Enteritis | 22 |
| Entero-colitis | 24 |
| Epilepsy | 3 |
| Erysipelas | 1 |
| Exhaustion | 2 |
| Fever, intermittent | 1 |
| " puerperal | 2 |
| " remittent | 2 |
| " scarlet | 11 |
| Fever typhoid | 19 |
| Gastritis | 3 |
| Gastro-enteritis | 10 |
| Hæmorrhage | 1 |
| Hæmoptysis | 1 |
| Heart, disease of | 7 |
| " fatty degeneration of | 2 |
| " organic disease of | 3 |
| " neuralgia of | 1 |
| " valvular disease of | 4 |
| Hydrocephalus | 14 |
| Inanition | 32 |
| Intemperance | 2 |
| Intussusception | 1 |
| Kidneys, Bright's disease of | 2 |
| " disease of | 2 |
| " inflammation of | 2 |
| Laryngitis | 1 |
| Liver, inflammation of | 1 |
| Lungs, congestion of | 3 |
| " hæmorrhage of | 1 |
| " œdema of | 1 |
| Manslaughter | 1 |
| Mania | 1 |
| " puerperal | 1 |
| Metro-peritonitis | 3 |
| Measles | 4 |
| Meningitis | 15 |
| " cerebro-spinal | 11 |
| " tubercular | 2 |
| Metritis | 1 |
| Myelitis | 1 |
| Old age | 12 |
| Paralysis | 4 |
| Peritonitis | 6 |
| Pneumonia | 15 |
| Pyæmia | 1 |
| Pyelitis | 1 |
| Rheumatism | 1 |
| Scrofula | 3 |
| Septicæmia | 2 |
| Synovitis | 1 |
| Syphilis | 1 |
| Syncope | 1 |
| Suicide | 6 |
| Tabes mesenterica | 12 |
| Teething | 13 |
| Tetanus | 3 |
| Uræmia | 1 |
| Uterus, hyperplasia of | 1 |
| Vitality, deficient | 1 |
| Whooping cough | 21 |
| Total | 986 |

Premature births, 10; Still births, 54. Total, 64.

COMPARISON.

Deaths in August, 1875, 986; in July, 1875, 1,174. Decrease, 188. Deaths in August, 1874, 1,220. Decrease, 234.

AGES.

| | | | |
|---|---|---|---|
| Under one year | 460 | Forty years to fifty | 42 |
| One year to two | 216 | Fifty " " sixty | 31 |
| Two years to three | 21 | Sixty " " seventy | 18 |
| Three " " four | 22 | Seventy " " eighty | 15 |
| Four " " five | 9 | Eighty " " ninety | 3 |
| Five " " ten | 20 | Ninety " " one hundred | 2 |
| Ten " " twenty | 24 | One hundred and upwards | 2 |
| Twenty " " thirty | 51 | | |
| Thirty " " forty | 50 | Total | 986 |

| | | | | | |
|---|---|---|---|---|---|
| White | 974 | Males | 500 | Married | 172 |
| Colored | 12 | Females | 486 | Single | 814 |
| Total | 986 | Total | 986 | Total | 986 |

NATIVITIES.

| | | | | | |
|---|---|---|---|---|---|
| Austria | 1 | England | 9 | Sweden | 4 |
| Belgium | 1 | Germany | 64 | Switzerland | 1 |
| Bohemia | 9 | Hungary | 1 | Wales | 1 |
| Canada | 4 | Ireland | 59 | West Indies | 1 |
| Native—Chicago | 169 | Italy | 1 | Unknown | 7 |
| Foreign, " | 528 | Norway | 7 | | |
| U. States, other parts | 110 | Nova Scotia | 1 | Total | 986 |
| Denmark | 3 | Scotland | 5 | | |

Deaths daily, 31 5-6. Mean thermometer, 68.6°. Rain fall, 3.29 inches.

MORTALITY BY WARDS.

| Wards. | No. Deaths. | Pop. in 1874. | Percentage. | Wards. | No. Deaths. | Pop. in 1874. | Percentage. |
|---|---|---|---|---|---|---|---|
| 1 | 4 | 5,725 | one death in 1,431 | 11 | 20 | 14,022 | one death in 701 |
| 2 | 4 | 4,830 | " " 1,207 | 12 | 25 | 16,792 | " " 672 |
| 3 | 22 | 14,861 | " " 675 | 13 | 25 | 17,892 | " " 716 |
| 4 | 21 | 15,361 | " " 732 | 14 | 38 | 16,720 | " " 440 |
| 5 | 33 | 20,078 | " " 609 | 15 | 180 | 45,545 | " " 253 |
| 6 | 113 | 35,916 | " " 318 | 16 | 56 | 21,922 | " " 448 |
| 7 | 104 | 31,722 | " " 305 | 17 | 54 | 20,777 | " " 385 |
| 8 | 72 | 29,143 | " " 404 | 18 | 49 | 21,392 | " " 437 |
| 9 | 42 | 31,654 | " " 754 | 19 | 12 | 4,677 | " " 389 |
| 10 | 19 | 17,385 | " " 915 | 20 | 13 | 8,995 | " " 692 |

Ratio of deaths to population in 1874, one death in 402.

| | | | |
|---|---|---|---|
| No. deaths in Wards | 906 | Manslaughter | 1 |
| Accidents | 35 | Police Station | 1 |
| County Hospital | 11 | St. Joseph's Hospital | 1 |
| Foundlings' Home | 15 | Suicides | 6 |
| Hospital Alexian Bros. | 4 | | |
| Hospital for Women and Children | 1 | Total | 986 |
| Mercy Hospital | 5 | | |

